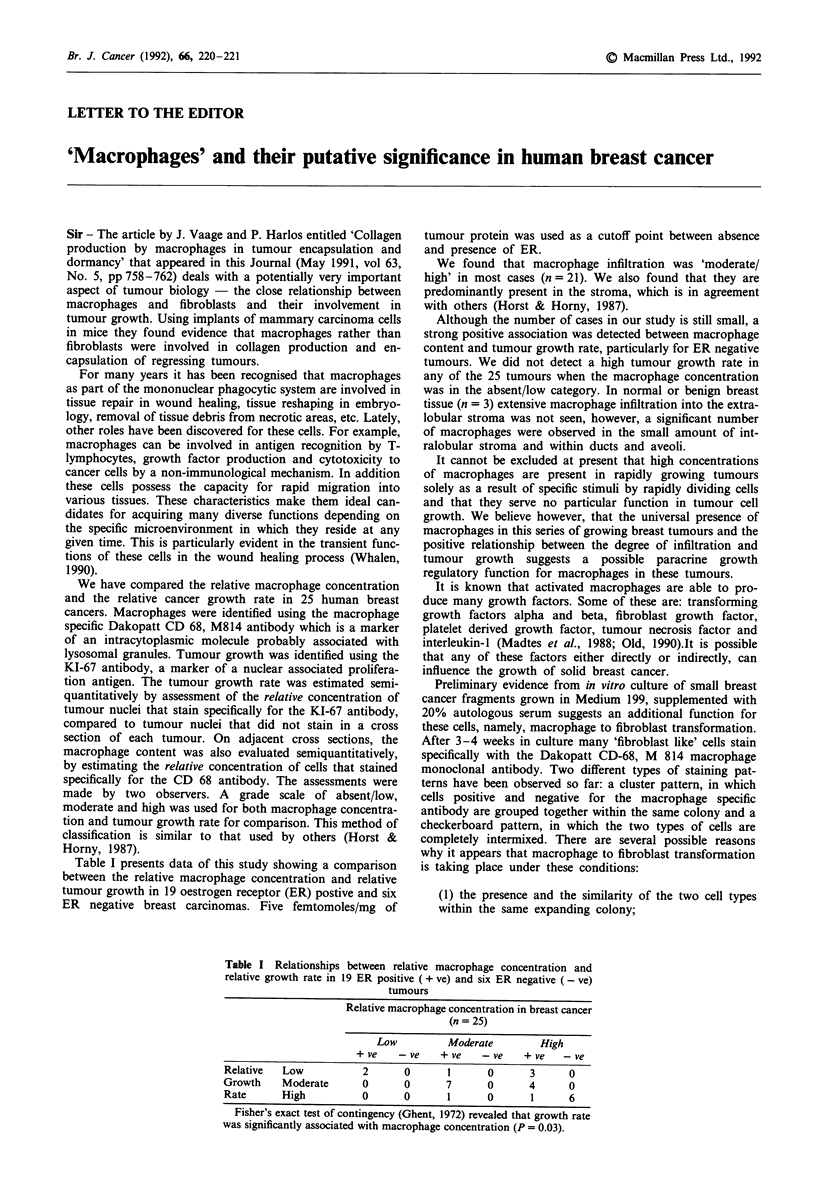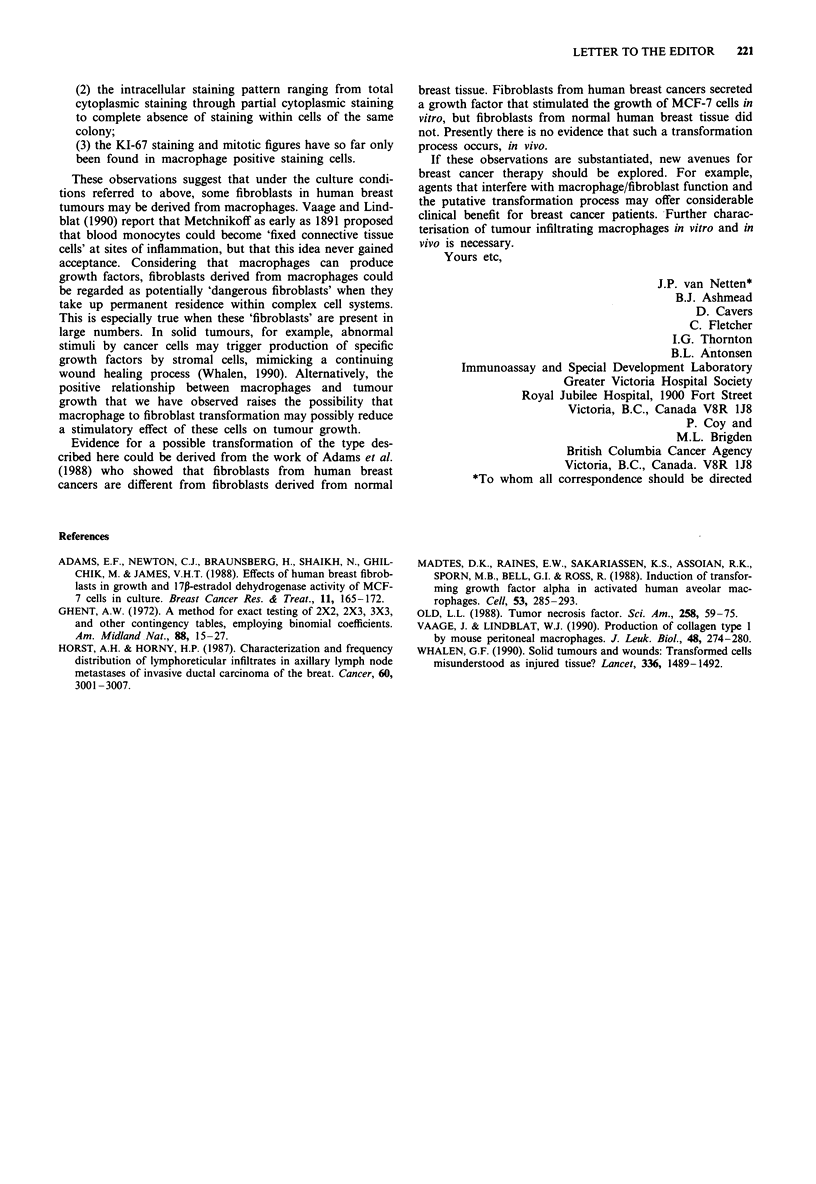# 'Macrophages' and their putative significance in human breast cancer.

**DOI:** 10.1038/bjc.1992.245

**Published:** 1992-07

**Authors:** J. P. van Netten, B. J. Ashmed, D. Cavers, C. Fletcher, I. G. Thornton, B. L. Antonsen, P. Coy, M. L. Brigden


Br. J. Cancer (1992), 66, 220-221 © Macmillan Press Ltd., 1992

### LETTER TO THE EDITOR

### 'Macrophages' and their putative significance in human breast cancer

Sir – The article by J. Vaage and P. Harlos entitled 'Collagen production by macrophages in tumour encapsulation and dormancy' that appeared in this Journal (May 1991, vol 63, No. 5, pp 758–762) deals with a potentially very important aspect of tumour biology — the close relationship between macrophages and fibroblasts and their involvement in tumour growth. Using implants of mammary carcinoma cells in mice they found evidence that macrophages rather than fibroblasts were involved in collagen production and encapsulation of regressing tumours.

For many years it has been recognised that macrophages as part of the mononuclear phagocytic system are involved in tissue repair in wound healing, tissue reshaping in embryology, removal of tissue debris from necrotic areas, etc. Lately, other roles have been discovered for these cells. For example, macrophages can be involved in antigen recognition by T-lymphocytes, growth factor production and cytotoxicity to cancer cells by a non-immunological mechanism. In addition these cells possess the capacity for rapid migration into various tissues. These characteristics make them ideal candidates for acquiring many diverse functions depending on the specific microenvironment in which they reside at any given time. This is particularly evident in the transient functions of these cells in the wound healing process (Whalen, 1990).

We have compared the relative macrophage concentration and the relative cancer growth rate in 25 human breast cancers. Macrophages were identified using the macrophage specific Dakopatt CD 68, M814 antibody which is a marker of an intracytoplasmic molecule probably associated with lysosomal granules. Tumour growth was identified using the KI-67 antibody, a marker of a nuclear associated proliferation antigen. The tumour growth rate was estimated semiquantitatively by assessment of the *relative* concentration of tumour nuclei that stain specifically for the KI-67 antibody, compared to tumour nuclei that did not stain in a cross section of each tumour. On adjacent cross sections, the macrophage content was also evaluated semiquantitatively, by estimating the *relative* concentration of cells that stained specifically for the CD 68 antibody. The assessments were made by two observers. A grade scale of absent/low, moderate and high was used for both macrophage concentration and tumour growth rate for comparison. This method of classification is similar to that used by others (Horst & Horny, 1987).

Table I presents data of this study showing a comparison between the relative macrophage concentration and relative tumour growth in 19 oestrogen receptor (ER) postive and six ER negative breast carcinomas. Five femtomoles/mg of tumour protein was used as a cutoff point between absence and presence of ER.

We found that macrophage infiltration was 'moderate/high' in most cases ($n = 21$). We also found that they are predominantly present in the stroma, which is in agreement with others (Horst & Horny, 1987).

Although the number of cases in our study is still small, a strong positive association was detected between macrophage content and tumour growth rate, particularly for ER negative tumours. We did not detect a high tumour growth rate in any of the 25 tumours when the macrophage concentration was in the absent/low category. In normal or benign breast tissue ($n = 3$) extensive macrophage infiltration into the extralobular stroma was not seen, however, a significant number of macrophages were observed in the small amount of intralobular stroma and within ducts and aveoli.

It cannot be excluded at present that high concentrations of macrophages are present in rapidly growing tumours solely as a result of specific stimuli by rapidly dividing cells and that they serve no particular function in tumour cell growth. We believe however, that the universal presence of macrophages in this series of growing breast tumours and the positive relationship between the degree of infiltration and tumour growth suggests a possible paracrine growth regulatory function for macrophages in these tumours.

It is known that activated macrophages are able to produce many growth factors. Some of these are: transforming growth factors alpha and beta, fibroblast growth factor, platelet derived growth factor, tumour necrosis factor and interleukin-1 (Madtes *et al.*, 1988; Old, 1990).It is possible that any of these factors either directly or indirectly, can influence the growth of solid breast cancer.

Preliminary evidence from *in vitro* culture of small breast cancer fragments grown in Medium 199, supplemented with 20% autologous serum suggests an additional function for these cells, namely, macrophage to fibroblast transformation. After 3–4 weeks in culture many 'fibroblast like' cells stain specifically with the Dakopatt CD-68, M 814 macrophage monoclonal antibody. Two different types of staining patterns have been observed so far: a cluster pattern, in which cells positive and negative for the macrophage specific antibody are grouped together within the same colony and a checkerboard pattern, in which the two types of cells are completely intermixed. There are several possible reasons why it appears that macrophage to fibroblast transformation is taking place under these conditions:

(1) the presence and the similarity of the two cell types within the same expanding colony;

**Table I** Relationships between relative macrophage concentration and relative growth rate in 19 ER positive (+ve) and six ER negative (−ve) tumours

| | | Relative macrophage concentration in breast cancer ($n = 25$) | | | | | |
|---|---|---|---|---|---|---|---|
| | | *Low* | | *Moderate* | | *High* | |
| | | *+ve* | *−ve* | *+ve* | *−ve* | *+ve* | *−ve* |
| Relative | Low | 2 | 0 | 1 | 0 | 3 | 0 |
| Growth | Moderate | 0 | 0 | 7 | 0 | 4 | 0 |
| Rate | High | 0 | 0 | 1 | 0 | 1 | 6 |

Fisher's exact test of contingency (Ghent, 1972) revealed that growth rate was significantly associated with macrophage concentration ($P = 0.03$).

(2) the intracellular staining pattern ranging from total cytoplasmic staining through partial cytoplasmic staining to complete absence of staining within cells of the same colony;

(3) the KI-67 staining and mitotic figures have so far only been found in macrophage positive staining cells.

These observations suggest that under the culture conditions referred to above, some fibroblasts in human breast tumours may be derived from macrophages. Vaage and Lindblat (1990) report that Metchnikoff as early as 1891 proposed that blood monocytes could become 'fixed connective tissue cells' at sites of inflammation, but that this idea never gained acceptance. Considering that macrophages can produce growth factors, fibroblasts derived from macrophages could be regarded as potentially 'dangerous fibroblasts' when they take up permanent residence within complex cell systems. This is especially true when these 'fibroblasts' are present in large numbers. In solid tumours, for example, abnormal stimuli by cancer cells may trigger production of specific growth factors by stromal cells, mimicking a continuing wound healing process (Whalen, 1990). Alternatively, the positive relationship between macrophages and tumour growth that we have observed raises the possibility that macrophage to fibroblast transformation may possibly reduce a stimulatory effect of these cells on tumour growth.

Evidence for a possible transformation of the type described here could be derived from the work of Adams *et al.* (1988) who showed that fibroblasts from human breast cancers are different from fibroblasts derived from normal breast tissue. Fibroblasts from human breast cancers secreted a growth factor that stimulated the growth of MCF-7 cells *in vitro*, but fibroblasts from normal human breast tissue did not. Presently there is no evidence that such a transformation process occurs, *in vivo*.

If these observations are substantiated, new avenues for breast cancer therapy should be explored. For example, agents that interfere with macrophage/fibroblast function and the putative transformation process may offer considerable clinical benefit for breast cancer patients. Further characterisation of tumour infiltrating macrophages *in vitro* and *in vivo* is necessary.

Yours etc,

J.P. van Netten*
B.J. Ashmead
D. Cavers
C. Fletcher
I.G. Thornton
B.L. Antonsen
Immunoassay and Special Development Laboratory
Greater Victoria Hospital Society
Royal Jubilee Hospital, 1900 Fort Street
Victoria, B.C., Canada V8R 1J8
P. Coy and
M.L. Brigden
British Columbia Cancer Agency
Victoria, B.C., Canada. V8R 1J8
*To whom all correspondence should be directed

### References

ADAMS, E.F., NEWTON, C.J., BRAUNSBERG, H., SHAIKH, N., GHILCHIK, M. & JAMES, V.H.T. (1988). Effects of human breast fibroblasts in growth and 17β-estradol dehydrogenase activity of MCF-7 cells in culture. *Breast Cancer Res. & Treat.*, **11**, 165–172.

GHENT, A.W. (1972). A method for exact testing of 2X2, 2X3, 3X3, and other contingency tables, employing binomial coefficients. *Am. Midland Nat.*, **88**, 15–27.

HORST, A.H. & HORNY, H.P. (1987). Characterization and frequency distribution of lymphoreticular infiltrates in axillary lymph node metastases of invasive ductal carcinoma of the breat. *Cancer*, **60**, 3001–3007.

MADTES, D.K., RAINES, E.W., SAKARIASSEN, K.S., ASSOIAN, R.K., SPORN, M.B., BELL, G.I. & ROSS, R. (1988). Induction of transforming growth factor alpha in activated human aveolar macrophages. *Cell*, **53**, 285–293.

OLD, L.L. (1988). Tumor necrosis factor. *Sci. Am.*, **258**, 59–75.

VAAGE, J. & LINDBLAT, W.J. (1990). Production of collagen type 1 by mouse peritoneal macrophages. *J. Leuk. Biol.*, **48**, 274–280.

WHALEN, G.F. (1990). Solid tumours and wounds: Transformed cells misunderstood as injured tissue? *Lancet*, **336**, 1489–1492.